# CytroCell: Valued Cellulose from Citrus Processing Waste

**DOI:** 10.3390/molecules26030596

**Published:** 2021-01-23

**Authors:** Antonino Scurria, Lorenzo Albanese, Mario Pagliaro, Federica Zabini, Francesco Giordano, Francesco Meneguzzo, Rosaria Ciriminna

**Affiliations:** 1 Istituto per lo Studio dei Materiali Nanostrutturati, CNR, via U. La Malfa 153, 90146 Palermo, Italy; antonino.scurria@ismn.cnr.it (A.S.); mario.pagliaro@cnr.it (M.P.); francesco.giordano@cnr.it (F.G.); 2 Istituto per la Bioeconomia, CNR, via Madonna del Piano 10, 50019 Sesto Fiorentino, Italy; lorenzo.albanese@cnr.it (L.A.); federica.zabini@cnr.it (F.Z.)

**Keywords:** cellulose, lemon, grapefruit, citrus processing waste, hydrodynamic cavitation, bioeconomy

## Abstract

Isolating cellulose from citrus processing waste without employing chemicals has so far been an unfulfilled goal of chemical research applied to the valorization of a widely available biowaste, annually totaling >100 million tonnes. We have applied hydrodynamic cavitation using a Venturi-type reactor for the extraction of all valued bioproducts of industrial citrus processing waste in water only, directly on a semi-industrial scale. After reporting the discovery of IntegroPectin in the soluble fraction of the aqueous extract, we now report the isolation of a cellulosic material in the water-insoluble fraction of cavitated lemon and grapefruit processing waste. Named “CytroCell”, the material is cellulose of low crystallinity, high porosity, good water holding capacity and good dispersibility in water. These properties open the route to mass-scale production of a useful functional material from a cheap and abundant biowaste.

## 1. Introduction

Citrus processing waste (CPW), namely citrus bagasse comprised of compressed peel, seeds and segmental membrane residual of the citrus juice industry, has long been identified as a potential source of multiple valued bioproducts, including cellulose [1,2]. Multiple chemical routes have been developed for the extraction of citrus cellulose from said by-product, generally starting from treatment with soda to remove lignin and hemicellulose, either with ethylenediaminetetraacetic acid as chelating agent [3] or followed by bleaching with 4% H_2_O_2_ under a basic condition at 90 °C [4]. Noting that the main use of CPW from orange and grapefruit juice production plants (over 10 million tonnes per year in the USA alone) is as low-value cattle feed obtained by energy-intensive drying of CWP, scholars in 2013 identified the integrated physico-chemical pretreatments of the biowaste, including high-speed grinding and treatment with caustic soda as the main challenges to the industrial development of the citrus biorefinery [5]. 

Beyond microcrystalline cellulose, waste orange peel has long been studied as a source of nanocellulose, namely a nanostructured material of exceptional mechanical (and thermal) properties ideally suited, if available at low cost and large amounts, for potentially numerous industrial applications [6], including carmaking using nanocellulose-based composites. Routes to nanocellulose derived from citrus processing waste include the use of enzymes [7], microwaves [8] and acids [9].

Nanocellulose has so far been industrially manufactured on small scale from bleached wood pulp in the form of nanofibrillated cellulose (also known as microfibrillated cellulose or cellulose nanofiber) after chemical delamination (fibrillation) of the cellulose fibers via TEMPO-mediated oxidation [9] (TEMPO is the 2,2,6,6-tetramethylpiperidine-1-oxyl radical that at buffered alkaline pH with hypochlorite as primary oxidant selectively oxidises the primary alcohol groups of the glucose units [10]). The nanomaterial is chiefly used in the paperboard and packaging industry to manufacture paperboard enhanced with microfibrillated cellulose in milk cartons for the dairy industry [11]. 

As explained by Guan and co-workers [6], biomass pretreatment to remove all non-cellulosic material (lignin and pectin) from lignocellulosic biomass—requiring numerous chemical and mechanical steps followed by nanocellulose extraction via acid hydrolysis, generating large amounts of wastewater, high energy consumption for a mechanical process, or long reaction time for enzymatic hydrolysis—has so far impeded the mass-scale production of an exceptionally versatile biomaterial. 

Perhaps the cleanest processes reported so far are the stepwise microwave hydrothermal treatment of dried depectinated orange peel from 120 °C to 180 °C to produce nanocellulose fibrils [8] and the autoclaving of lime processing waste at 110–130 °C to remove hemicellulose and pectin followed by high shear (at 20,000 rpm for 15 min) and high-pressure (at 40 MPa for 5 passes) homogenizing [12]. Though demonstrated at the lab scale at low dried peel load (1 g of dried peel mixed with 70 g of distilled water), the nanocellulose obtained via the microwave hydrothermal treatment of dried depectinated orange peel had a high water retention capacity varying between 16 and 20 g_H_2_O_ g^−1^ but was deeply colored brown due to the Maillard reaction products formed from the degradation/caramelization of sugars and their further reaction with residual proteins at the high temperatures employed [8]. 

Hydrodynamic cavitation (HC) is increasingly applied in the extraction of natural products from multiple biomass sources [13]. In 2019, our teams applied a method to extract the main bioproducts of orange processing waste directly on a semi-industrial scale, i.e., processing 42 kg of wet waste orange peel (WOP) obtained from the citrus juice industry in 120 L water [14]. Focusing our initial attention on the water-soluble products resulting from the extraction, we isolated a unique form of pectin (named “IntegroPectin”) with a low degree of esterification (17%) and containing plentiful amounts of co-extracted orange flavonoids hesperidin and naringin, raising global interest in HC as an efficient and highly scalable hesperidin production method for the prevention or treatment of COVID-19 [15]. Shortly afterward, we extended the method on the same semi-industrial scale to lemon and grapefruit processing waste, isolating a lemon pectin showing exceptional antioxidant [16] and good antibacterial [17] properties and a grapefruit pectin showing even higher and broad-spectrum bactericidal action [18]. 

The only investigation carried out on the insoluble, cellulose-rich fraction obtained from HC of waste orange peel showed that the HC process was able to effectively increase the methane generation from the solid residues of the WOP material, with a clear increasing trend during the hydrocavitation process up to the full exploitation of the respective biochemical methane potential (BMP), reaching +8% of the theoretical BMP for the hydrocavitated solid sample obtained after 270 min of cavitation [14]. Now, we report the first structural investigation of the cellulose-rich fraction isolated from the HC-based extraction of lemon and grapefruit industrial processing waste. This new biomaterial (see below) is named herein “CytroCell”. 

## 2. Results and Discussion

### 2.1. Textural Properties

**Figure 1:**
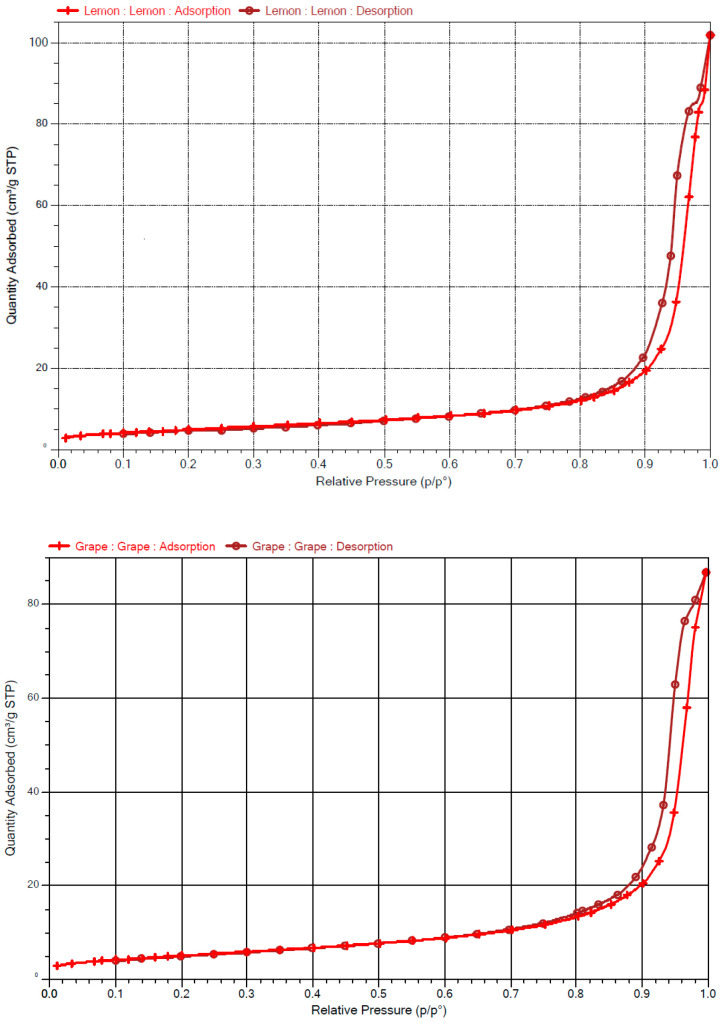
Low-temperature N_2_ adsorption-desorption isotherms for lemon (top) and grapefruit (bottom) CytroCell.

The physisorption isotherms showing N_2_ adsorption at 77 K and at sub-atmospheric pressures in Figure 1 show that both lemon and grapefruit CytroCell are mesoporous materials (*caveat*: the high electrostatic charge on the surface of both celluloses is allowed to solely use an aggregate rather than a powdered specimen to carry out the experiments). The shape of the isotherms intermediate between type IV (irreversible type IV isotherm characteristic of mesoporous solids, with pore size >2 nm) and type II (reversible type II isotherm characteristic of macroporous solids with pore size >50 nm). The hysteresis loop of the resulting Type IVa isotherm for mesoporous materials with pore size >4 nm, located in the multilayer range of the isotherm, is associated with said capillary condensation [19].

Comparison of the pore size and specific pore volume obtained via the classical pore size model developed by Barret, Joyner and Halenda (BJH) from the adsorption and desorption branches of the isotherms shows similar values for both celluloses, with the exception of the pore size of lemon CytroCell measured on the adsorption branch, which is 2 nm larger than the pore size measured on the desorption branch (entries 1 and 2 in Table 1). 

**Figure 2:**
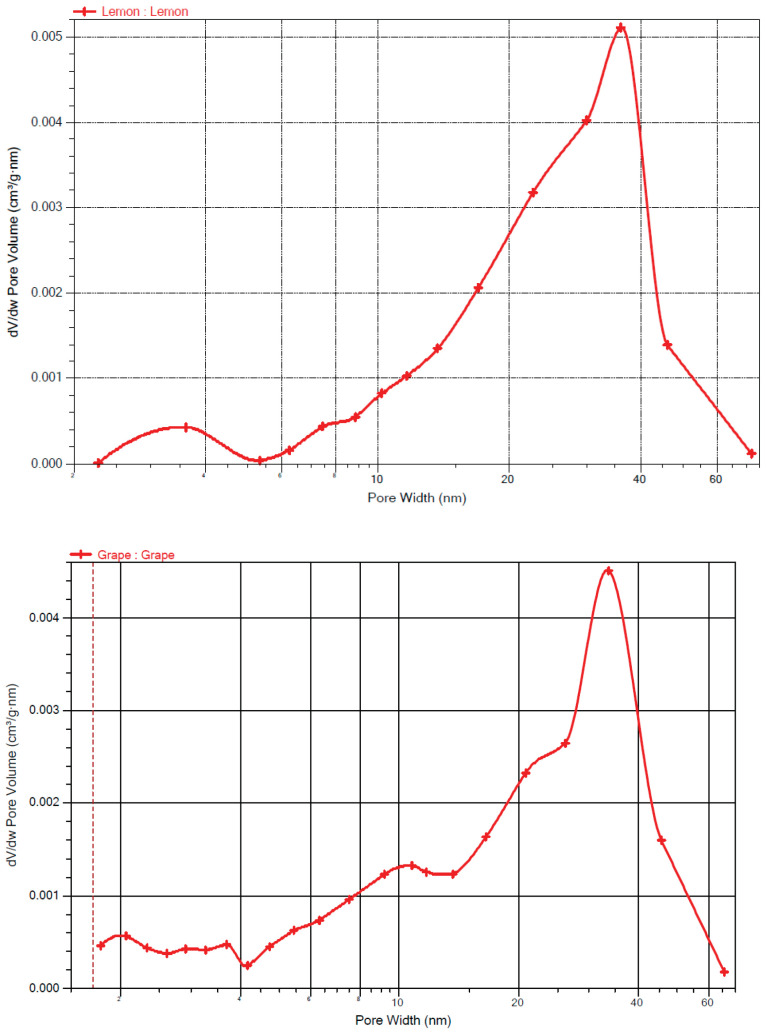
Pore size distribution for lemon (top) and grapefruit (bottom) CytroCell from the respective BJH isotherm desorption branch.

Coupled with the shape of the isotherms, these results indicate that the citrus structure of CytroCell includes large and open mesopores with a broad size distribution as observed in the case of certain tropical wood aerogels [20]. The pore size from the desorption branch, indeed, corresponds to the aperture (entrance) size of the pore, whereas the pore size distribution (Figure 2) obtained from the adsorption branch of the isotherm corresponds to the size of the cavity [21]. 

Like the aforementioned tropical wood aerogels [20], the very narrow hysteresis loops in Figure 1, with the adsorption and desorption branches almost vertical and nearly parallel above 0.9 relative pressure, indicates the relatively large outer surface for both celluloses. 

### 2.2. IR Spectroscopy

**Figure 3:**
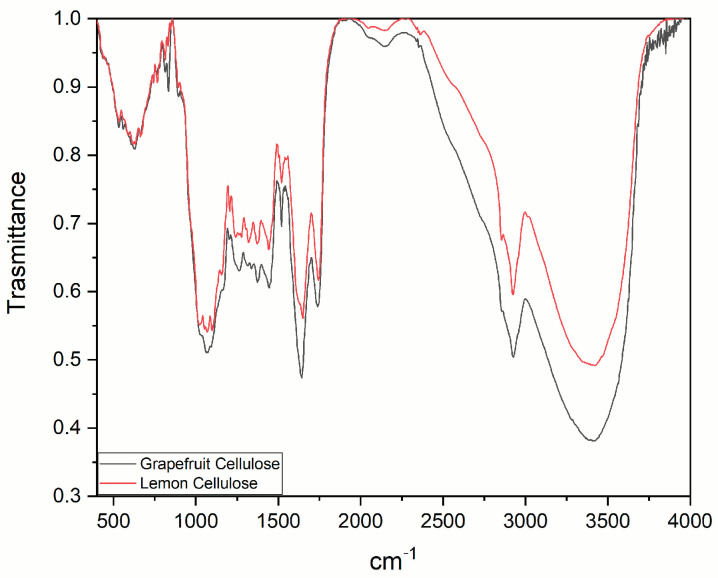
IR spectra for for lemon (red) and grapefruit (black) CytroCell.

The FTIR spectra in Figure 3 display all the characteristic cellulose peaks (Table 2), including those in the fingerprint region of 1000–1200 cm^−1^ [22,23]. 

The broad band around 3330–3400 cm^−1^ corresponds to the stretching vibrations of O-H bonds of free and bound water and O-H bonds of hydroxyl groups [24]. The bands at 2850–2925 cm^−1^ are due to the stretching vibrations of the C-H bonds in the saturated carbon rings of polysaccharides and to the stretching vibrations of these bonds in CH_2_ groups [24]. The signal at 1160 cm^−1^ wavelength derives from the stretching vibration of the C-O-C β-(1-4)-glycosidic bond as well as to the stretching vibrations of C-O bonds in saturated six-membered rings, whereas the signal at 897 cm^−1^ is due to νC-O-C at the same glycosidic linkage from the amorphous region of cellulose [25].

**Scheme 1:**
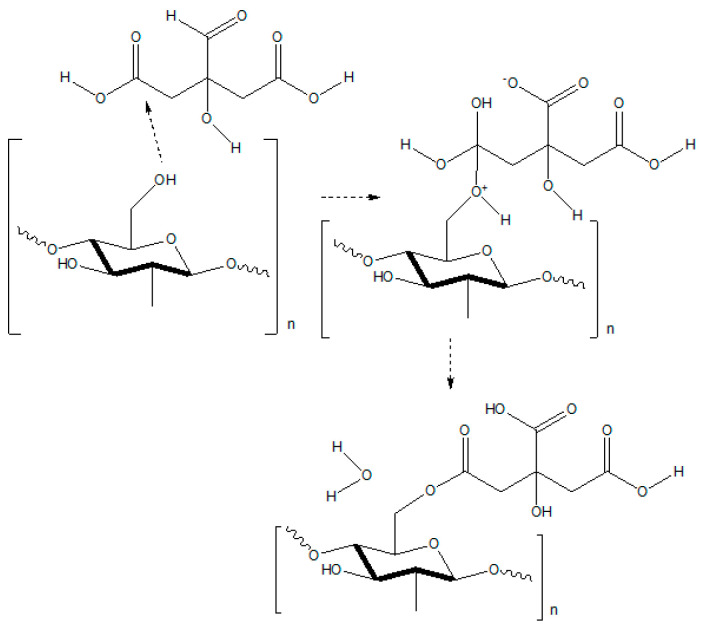
Esterification of cellulose primary alcohol groups and citric acid.

The bands at 1650 cm^−1^ and at 1730 cm^−1^ are not due to pectin, which is efficiently separated from the lignocellulosic biomass and solubilized in water during the cavitation-based extraction process, but rather to the free and esterified carboxyl groups of cellulose esterified with citric acid upon reaction of the citric acid residual in the wet lemon (and in the wet grapefruit) bagasse and the primary alcohol groups of cellulose (Scheme 1) [26]. 

Remarkably, the same peaks were observed and ascribed to the same esterified groups by scholars in Turkey analyzing a lemon resin obtained by boiling small pieces of lemon fruit [27], as well as by Brazilian scholars producing nanocellulose by reacting wood cellulose pulp with aqueous citric acid at 120 °C [28]. 

We make the hypothesis that the extreme conditions created in the imploding cavitation bubbles ease the reaction of citric acid with the primary hydroxyl group of cellulose chains to form an ester bond like what happens in the latter process eventually affording partly esterified cellulose. Citric acid also plays a crucial role in converting the residual lignin in the waste citrus peel by creating an catalytic environment [8] similar to that of the microwave-assisted extraction able to hydrolyze lignin via proton transfer, β-elimination and ester/ether cleavage [8].

### 2.3. X-ray Diffraction

**Figure 4:**
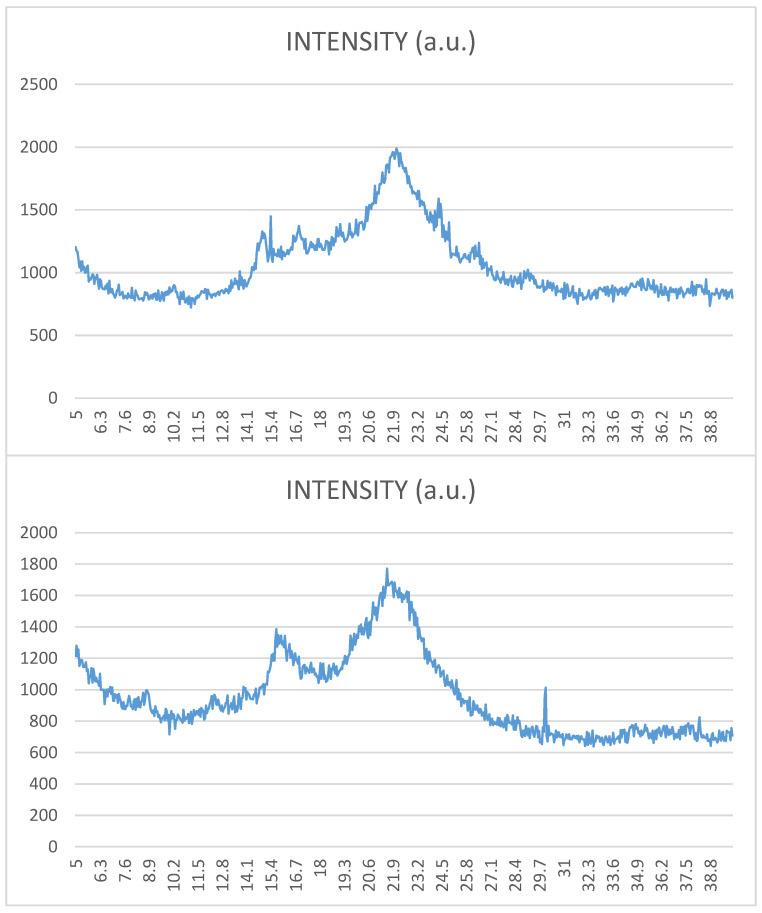
XRD spectra for lemon (top) and grapefruit (bottom) CytroCell.

The XRD spectra in Figure 4 display all the characteristic diffraction peaks of cellulose from citrus waste with the 16.4° peak (101 plane), which can be separated into amorphous and crystalline portions, an amorphous region between 16.4° and 18.0°, and the highest peak at 22.4° corresponding to diffraction from the 002 plane [8,29]. 

The crystallinity index (CI) readily calculated using the Segal equation (Equation (1)), namely the ratio between the difference between the 002-peak intensity (*I*_002_) and the intensity *I*_am_ of diffraction 2*ϑ* = 18° corresponding to the proportion of the amorphous cellulose [29]:

CI = (*I*_002_ − *I*_am_)/*I*_002_ (1)

Using the intensity arbitrary units from the diffractograms displayed in Figure 4, the aforementioned equation returns exceptionally low levels values for the CI of 0.33 for lemon CytroCell (Equation (2)) and 0.36 for grapefruit CytroCell (Equation (3)):

CI (lemon CytroCell) = (1650 − 1100))/1650 ≃ 0.33(2)

CI (grapefruit CytroCell) = (1950 − 1250)/1950 ≃ 0.36(3)

This points to a key role of citric acid (far more abundant in lemon) during the hydrodynamic cavitation of the wet industrial waste of the citrus juice industry, analogous to what was observed in the conversion of the key citrus terpenes limonene and linalool [30]. For comparison, the crystallinity index of orange bagasse nanocellulose obtained upon enzymatic hydrolysis of bacterial cellulose is 0.63 [7], still significantly lower than that of cotton nanofibers, which have a CI of 0.78 [31]. Causing such a low CI value, we hypothesize, are the extreme pressure and temperature local conditions determined by the implosion of the cavitation bubbles. This defibrillates the cellulose fibers and alters the crystalline morphology of native citrus cellulose. Subsequent microscopic investigation will investigate the validity of this hypothesis.

Like what was observed for citrus depectinated nanocellulose obtained via microwave-assisted extraction [6], peaks at about 15°, 24° and 30° deriving from CaC_2_O_4_ were observed in the XRD spectra, particularly in the case of grapefruit CytroCell (Figure 4, bottom). Calcium oxalate crystals are ubiquitous in many plants (in widely different parts of the plant, from leaves to roots) where they exert multiple functions against both abiotic (drought, nutrient deprivation, metal toxicity) and biotic (pathogens and herbivore) stress factors [32].

### 2.4. Zeta Potential

The results of the zeta potential, an indirect measure of the net charge of particles in suspension, which indicates the stability of a suspension (the larger the ζ potential absolute value, the more stable the colloidal dispersion), displayed in Table 3 point to a negative value approaching −30 mV for lemon CytroCell and −23 mV for grapefruit CytroCell.

In agreement with guidelines classifying nanoparticle dispersions derived from the Derjaguin, Landau, Verwey and Overbeek theory of colloids, this indicates that lemon CytroCell nanoparticles are highly stable (ζ ˃ ±30 mV), whereas grapefruit CytroCell particles (±20 < ζ < 30 mV) are moderately stable [33]. These values of the potential are close to those of wood nanocellulose obtained after the hydrothermal treatment of commercial cellulose with citric acid for 1.5 h (−36.5 mV [28]), ascribed to the presence of anionic carboxyl groups at the surface of nanocellulose, imparting increased electrostatic repulsion and thus enhanced stability of the colloidal suspension.

### 2.5. Water Holding Capacity

We measured the water holding (or retention) capacity (WHC) for both grapefruit and lemon CytroCell by leaving for 30 min a sample of mildly dried CytroCell with distilled water in a centrifuge tube. After centrifugation, the water retention capacity of lemon CytroCell was 8 g_water_/g_cell_, whereas that of grapefruit CytroCell was 5 g_water_/g_cell_. The water holding (or retention) capacity (of cellulose is a key parameter affecting the applications of cellulose in the food, pharmaceutical and cosmetic industries. In general, the WHC increases with increasing fiber length. For example, for powdered cellulose (70% crystalline and 30% amorphous), the WHC quickly increases between 4 g_water_/g_cell_ and 9.5 g_water_/g_cell_ for fibers between 35 μm and 110 μm, whereas it remains virtually constant for fibers shorter than 35 μm and longer than 110 μm [34]. 

**Figure 5:**
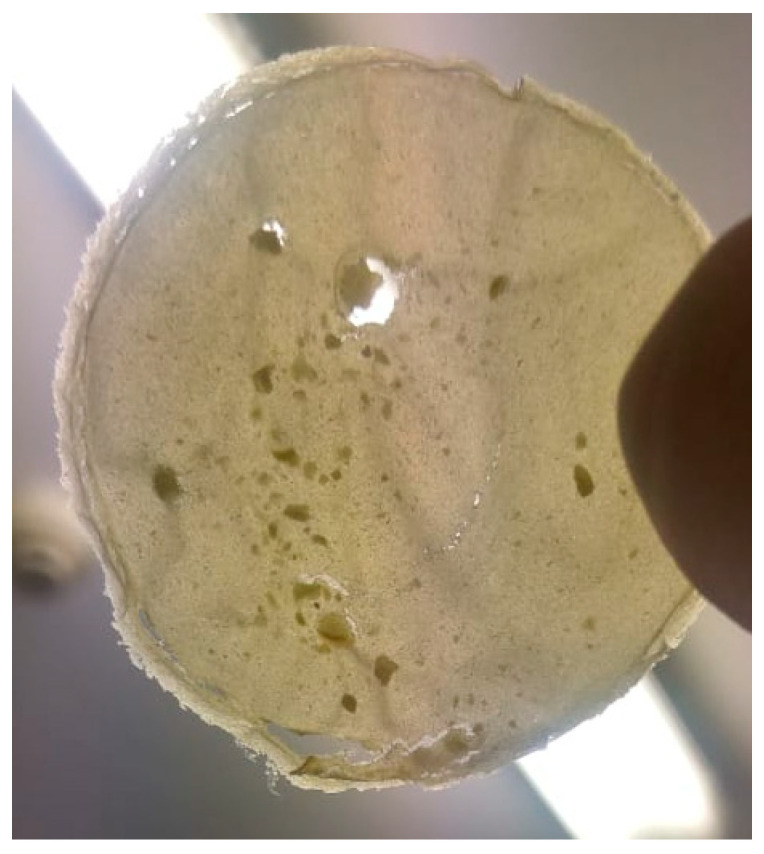
Disc of lemon CytroCell obtained upon mild drying of an aqueous paste.

Showing the large applicative potential of these new nanocelluloses due to their WHC, Figure 5 shows a disc of lemon CytroCell readily obtained upon mildly drying a dispersion of the material in water overnight at 35 °C. The porous and fibrous nature of the lightweight biomaterial is readily recognized. With nonuniform distribution of composition materials, the film is displayed only to demonstrate the ease with which the CytroCell aggregates forming a rigid film of significant thickness and strength. Subsequent work will use purified CytroCell to shape films of uniform size and composition reporting the mechanical and thermal properties for both CytroCell materials.

## 3. Materials and Methods

### 3.1. Materials Preparation

The raw materials (integral aqueous extracts of lemon and grapefruit bagasse) were obtained by means of hydrodynamic cavitation processes lasting 60 min as in previous experiments [14]. The HC extraction device included a closed hydraulic loop (total volume capacity around 230 L), a centrifugal pump (Lowara, Vicenza, Italy, ESHE 50–160/75) with 7.5 kW nominal mechanical power and rotation speed of 2900 rpm and a Venturi-shaped sealed reactor to minimize the loss of the lemon and grapefruit processing waste’s volatile components [16]. In detail, 34 kg of fresh waste lemon peel obtained from organically grown Siracusa lemons (*Citrus limon*, cultivar “femminello”) by an in-line extractor at a juice factory kindly donated by a citrus company based in Sicily were first ground in ice with an electric blender and then added along with 120 L of tap water to the HC device. The reactor was closed and cavitation started. The whole process lasted 60 min and consumed 6.70 kWh of electric energy, thus the specific consumed energy was 0.22 kWh per kg of fresh lemon processing waste. After completion, the liquid phase was collected and lyophilized to obtain the lemon IntegroPectin [16]. Prior to lyophilization, the insoluble cellulosic material in the cavitated aqueous mixture was filtered using a Büchner filter with Whatman ashless filter paper, grade 589/3. The aqueous phase was used to isolate IntegroPectin via lyophilization [16] while CytroCell cellulose was isolated from the filtered wet solid phase. A filtrate sample (50 g) including plentiful adsorbed water was mixed with 150 mL of distillate water under fast stirring (470 rpm) for 30 min using a KS 260 control flat shaker (IKA-Werke, Staufen, Germany). The washed material was further refined by pressing the cellulosic paste through the mesh of a laboratory test sieve (aperture of 212 µm, Endecotts, London, Great Britain) using a flat glass specimen. The refined material was dried in an oven at 35 °C for 20 h. The amount of isolated dried lemon and grapefruit CytroCell was 1.2 g and 1.7 g, respectively. 

### 3.2. Water Holding Capacity

To measure the water retention capacity, a sample of dried CytroCell (1 g) was mixed with 15 mL of deionized water and poured into two 20 mL centrifuged tubes. The two samples were stirred for 10 min and then centrifuged at 3000 rpm for 30 min using an Allegra X-22R benchtop centrifuge (Beckman Coulter, Palo Alto, CA, USA). After centrifugation, the supernatant aqueous phase was removed and the wet cellulose was weighed. 

### 3.3. FT-IR Analyses

**Figure 6:**
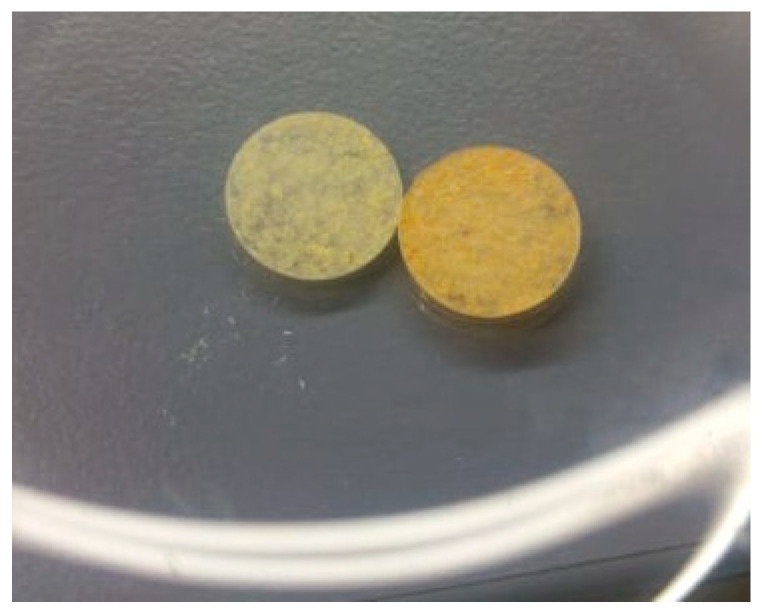
Pellets of lemon (yellow) and grapefruit (orange) CytroCell dispersed in KBr.

The IR analyses were performed with an ALPHA compact FT-IR spectrometer (Bruker Optics, Billerica, MA, USA). Each time, a sample of a few mg of lemon or grapefruit CytroCell cellulose in powder form was mixed with ultrapure KBr (FT-IR grade, ≥99% trace metals basis, Sigma-Aldrich, St. Louis, MI, USA). A small amount (ca. 20 mg) of CytroCell was mixed with an excess of KBr powder (ca. 150 mg) and ground using a pestle in an agate mortar to form a uniform mixture. A Specac Mini-Pellet laboratory hydraulic press was used for the preparation of high-quality 7 mm KBr pellets for transmission FTIR (Figure 6) applying a 12 t weight for 5 min.

### 3.4. XRD Analysis

The samples were analyzed by a D5005 X-ray diffractometer (Bruker AXS, Karlsruhe, Germany) operating at 40 kV and 30 mA to obtain the diffraction profile at 0.15°/min acquisition rate over a 5.0°–40.0° 2*θ* range. The X-ray radiation was generated via a copper (Kα) anode and made monochromatic via the instrument’s secondary monochromator. 

### 3.5. Nitrogen Physisorption Measurements 

The nitrogen adsorption isotherms were obtained using an ASAP 2020 Plus surface area and porosimetry system (Micromeritics Instrument Corporation, Norcross, GA, USA) equipped with the ASAP 2020 Plus Version 1.03 software. An aggregate sample of CytroCell (110.5 mg for grapefruit CytroCell and 93.5 mg for lemon CytroCell) inserted in a glass burette was first degassed by heating it for 10 min with a heating ramp rate of 10 °C/min. The analysis was carried out at 77.423 K with a 10 s equilibration interval. 

### 3.6. Disc Preparation

The same hydrated sample obtained after prolonged contact of 200 mg grapefruit or lemon CytroCell cellulose with water after centrifugation in the WRC test was added with 5 mL distillate water. After short homogenization using a vortex shaker (Vortex 1, IKA-Werke, Staufen, Germany), the mixture was dried in an oven at 35 °C for 20 h.

### 3.7. Zeta potential 

The zeta potential was determined at 25 °C with a Zetasizer Nano ZS analyzer (Malvern Panalytical, Malvern, Great Britain) using a laser wavelength of 633 nm to measure the speed of particle movement using laser Doppler electrophoresis. A suspension of CytroCell (20 mg) in 10 mL millQ water was employed in the measurements carried out in a DTS1070 cell. The zeta potential reported is the average of three measurements, which returned very similar results.

## 4. Conclusions

We have discovered a new form of citrus cellulose of low crystallinity index, large open mesoporosity, good water retention capacity and dispersibility in water, which can be easily synthesized in large amounts by hydrodynamic cavitation of citrus processing biowaste. Named “CytroCell”, this new form of cellulose studied herein originating from lemon and grapefruit bagasse has ultralow crystallinity of about 0.35, large mesoporosity (pore size close to or even exceeding 25 nm) and good water holding capacity approaching almost 10 g_water_/g_cell_ in the case of lemon CytroCell). Part of the cellulose fibrils, we argue in this study, are esterified with citric acid, abundant in the wet residue of lemon and grapefruit industrial juice production used as raw material. The biomaterial obtained from the food industry waste rather than from wood pulp is suitable for a wide variety of applications. The large pore diameter around 24 nm for both celluloses, for example, makes them suitable for catalysis (as metal support), separation (chromatography) and adsorption (decontamination) using an intrinsically sustainable biomaterial.

Carried out in water only, the physical production route does not require any pre-treatment of citrus processing waste and does not use acid, alkali, chemical oxidants or enzymes, thereby establishing an intrinsically green production route for cellulose starting from biobased waste available worldwide in over 110–120 million tonnes [35] using an easily scalable and low operating cost process increasingly employed for the extraction of numerous natural products [13,14]. The technology meets all six principles of Green Extraction of natural products [36]: i) use of renewable, plentiful plant resources; ii) solvent-free—water is the only solvent; iii) low energy consumption; iv) zero waste; v) reduced unit operations in favor of safe, robust and scalable and easily controllable processes (only two operations (i.e., HC processing and mechanical separation); vi) aims for a non-denatured and biodegradable extract without contaminants (absence of additives, water and raw materials are the only ingredient), while the HC-based process does not denaturate the bioactive compounds.

## Figures and Tables

**Table 1:** Specific pore volume and pore size for lemon and grapefruit CytroCell.

Entry	Sample	Specific Pore Volume (cm³/g)	Pore Size (nm)
**1**	Lemon CytroCell	0.137 ^a^	29.49 ^a^
**2**	Lemon CytroCell	0.137 ^b^	27.34 ^b^
**3**	Grapefruit CytroCell	0.117 ^a^	23.63 ^a^
**4**	Grapefruit CytroCell	0.125 ^b^	23.96 ^b^

^a^ From the Barret, Joyner and Halenda (BJH) adsorption branch; ^b^ from the BJH desorption branch.

**Table 2:** Selected absorption bands and corresponding vibrational modes of chemical groups in cellulose in the IR absorption spectrum of cellulose ^a^ (Adapted from Refs. [22] and [23], CC BY license).

Wavenumber of Absorption Bands (cm^−1^)	Chemical Group
3300–3400	νOH covalent bond, hydrogen bonding
2850–2925	νCH, νCH_2_
1641	absorbed water (hydrogen-bonded)
1428	δCH_2_ (symmetric) at C-6; crystalline region
1236	δCOH in plane at C-6
1160	νCOC at β-glycosidic linkage
1104	ν(C-O-C) in ring
1030	νCO at C-6
897	νCOC at β-glycosidic linkage, amorphous region
662	δCOH out of plane

^a^ ν = stretching, δ = bending.

**Table 3:** ζ potential for lemon and grapefruit CytroCell obtained using hydrodynamic cavitation.

Material	Zeta Potential (mV)
Lemon CytroCell	−29.5
Grapefruit CytroCell	−22.67

## Data Availability

All Data available by contacting the corresponding authors.

## Sample Availability

Samples of the compounds are available from the authors.
